# PSTPIP2 regulates synovial macrophages polarization and dynamics via ERβ in the joint microenvironment

**DOI:** 10.1186/s13075-022-02939-y

**Published:** 2022-11-02

**Authors:** Yao Yao, Xiaoyu Cai, Meng Zhang, Xiao Zhang, Fujia Ren, Yan Zheng, Weidong Fei, Mengdan Zhao, Caihong Zheng

**Affiliations:** 1 Department of Pharmacy, Women’s Hospital, Zhejiang University School of Medicine, Hangzhou, 310006 China; 2 Department of Clinical Pharmacology, Key Laboratory of Clinical Cancer Pharmacology and Toxicology Research of Zhejiang Province, Affiliated Hangzhou First People’s Hospital, Cancer Center, Zhejiang University School of Medicine, Hangzhou, 310006 China; 3 Department of Pharmacy, Hangzhou Women’s Hospital (Hangzhou Maternity and Child Health Care Hospital), Hangzhou, China; 4 Department of Geriatrics, Zhejiang Provincial People’s Hospital, Hangzhou, China

**Keywords:** Synovial macrophages, PSTPIP2, Rheumatoid arthritis, Polarization, Dynamics, Estrogen receptor

## Abstract

**Background:**

The cytoskeletal protein, PSTPIP2, is associated with inflammation and is predominantly expressed in macrophages. Previous data have shown that PSTPIP2 inhibits articular bone damage in arthritic rats. The aim of this study is to explore the molecular mechanism of PSTPIP2’s resistance to bone erosion.

**Methods:**

In the current study, peripheral blood and surgically excised synovial tissue from RA patients, DBA/1 mice, *Pstpip2*^Cre^R26-*ZsGreen* reporter mice, and *Esr2*^fl/fl^/*Adgre*-Cre tool mice were used for in vivo studies. Adeno-associated viral vector was used to overexpress PSPTIP2 protein in vivo.

**Results:**

We found that The level of PSTPIP2 in synovial macrophages is negatively correlated with RA disease activity, which is mediated by synovial macrophages polarization. PSTPIP2^hi^ synovial macrophages form a tight immunological barrier in the lining layer. Notably, the ability of PSTPIP2 to regulate synovial macrophages polarization is dependent on ERβ. Additionally, PSTPIP2 regulates the dynamics of synovial macrophages via ERβ.

**Conclusions:**

Together, this study reveals that PSTPIP2 regulates synovial macrophages polarization and dynamics via ERβ to form an immunological barrier (F4/80^+^PSTPIP2^hi^ cell-enriched zone) for the joints. Thus, local modulation of PSTPIP2 expression in the joint microenvironment may be a potential strategy for controlling bone erosion in rheumatoid arthritis.

**Graphical Abstract:**

PSTPIP2 regulates synovial macrophages polarization and dynamics via ERβ to form F4/80^+^PSTPIP2^hi^ cellular barrier in joint microenvironment.
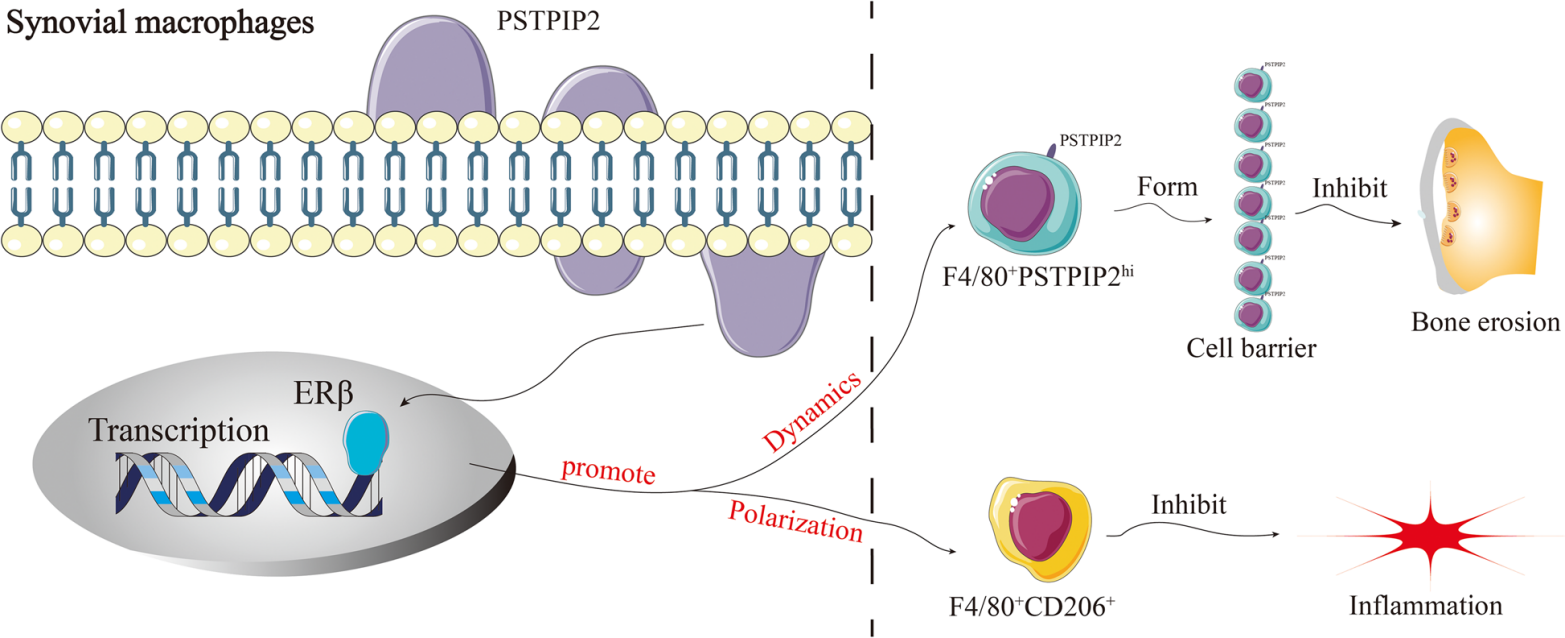

**Supplementary Information:**

The online version contains supplementary material available at 10.1186/s13075-022-02939-y.

## Background

Rheumatoid arthritis (RA) is an inflammatory and autoimmune disease characterized by synovial inflammation and bone erosion [1, 2]. The synovial inflammation and bone erosion in RA can cause joint damage, which in turn affects normal joint function and ultimately leads to limited mobility [3]. Currently, clinical treatment of RA relies on anti-inflammatory or anti-rheumatic drugs to suppress synovial inflammation and control the disease activity. However, the specificity of existing drugs is poor, and most of them treat the symptoms but not the root cause [4]. Therefore, it is important to explore the effective treatment strategies specific to RA for synovial inflammation and bone damage.

Proline-serine-threonine phosphatase interacting protein 2 (PSTPIP2), also known as macrophage actin-associated tyrosine phosphorylated protein or MAYP, is associated with autoinflammation [5] and mainly expressed in macrophages [6, 7]. Previous studies have shown that mutations in the *Pstpip2* gene in mice cause inflammatory macrophage disease [7, 8]. Our previous study demonstrated that overexpression of PSTPIP2 in the knee joint alleviated the inflammatory response and bone damage in arthritic rats [9].

In this study, we aimed to investigate the molecular mechanisms by which PSTPIP2 inhibits bone erosion. This study explored the molecular mechanisms by which PSTPIP2 regulates synovial macrophage activity in bone erosion from clinical issues. In brief, we found that PSTPIP2 regulates synovial macrophages polarization and dynamics via ERβ to form an immunological barrier (F4/80^+^PSTPIP2^hi^ synovial macrophages enriched area) for the joint. Thus, PSTPIP2 may be a specific target for the future treatment of RA. Local modulation of PSTPIP2 expression in the articular cavity in the early stages of RA may be a novel strategy for the treatment of RA.

## Materials and methods

### Human samples

Synovial tissues and peripheral blood from RA patients and acute trauma patients were obtained with fully informed consent. Inclusion and exclusion criteria for rheumatoid arthritis were performed according to the American Rheumatism Association rheumatoid arthritis classification criteria [10]. Acute trauma patients should have no history of arthritis. The rheumatoid factor (RF), antistreptolysin (ASO) and DAS28 scores of RA patients were derived from clinical sources. Participants' age, body mass index (BMI), alcohol consumption, smoking status, presence of common chronic conditions (e.g., hypertension, diabetes, and osteoarthritis), and medication use (e.g., angiotensin receptor blockers, metformin, and NSAIDs), did not differ significantly between the two groups (Supplementary Table 1).

### Mice

Five- to 8-week female DBA/1JGpt mice were purchased from GemPharmatech Co., Ltd. (Nanjing, China). *Esr2*^fl/fl^ mice (control) and *Esr2*^fl/fl^/*Adgre*-Cre mice (F4/80^+^ cell-specific knockout of *Esr2* gene) were purchased from GemPharmatech Co., Ltd. (Nanjing, China). *Pstpip2*^Cre^R26-*ZsGreen* reporter mice were purchased from Shanghai Model Organisms Center, Inc. (Shanghai, China).

### Induction of collagen-induced arthritis

Collagen-induced arthritis (CIA) was induced and assessed as an experimental procedure [11]. Briefly, the time of the primary immunization was defined as day0, and the secondary immunization was performed on day21. Each mouse was injected with 0.1 ml Freund’s complete adjuvant (containing 4mg/ml BCG and 2mg/ml chicken type II collagen) at multiple points on the root of the tail and back. Arthritis score and weight of mice were measured and recorded from day28 onwards. Arthritis scoring criteria: 0: normal, no joint redness or swelling; 1: mild, slight redness and swelling of the ankle or wrist, or obvious redness and swelling of the fingers; 2: moderate, redness and swelling of the ankle or wrist; 3: severe redness and swelling of the entire paw; and 4: severe redness and swelling including multiple joints. The cumulative values for the three paws were calculated as the total clinical arthritis score for each mouse, with a maximum score of 16 for each mouse. Day 35 is the peak of joint inflammation and day56 is the start of remission.

### In vivo treatments and cell culture

Animal experiments were performed in three batches. The first grouping was: Normal control, *Pstpip2*^Cre^R26-*ZsGreen*, and Wild. These mice were sacrificed on day 35 or day 70. Mice were anesthetized by intraperitoneal injection of sodium pentobarbital and then dislocated to death. Normal control mice did not receive any treatment and did not induce CIA. *Pstpip2*^Cre^R26-*ZsGreen* reporter mice and Wild mice were induced to CIA. The second grouping was: CIA, AAV-empty, and AAV-PSTPIP2. These mice were sacrificed on day 42. Mice were anesthetized by intraperitoneal injection of sodium pentobarbital and then dislocated to death. The knee cavities of mice in the AAV-PSTPIP2 group were injected with adeno-associated virus loaded with a plasmid overexpressing the *PSTPIP2* gene on day22. Mice in the AAV-empty group were injected with empty adeno-associated virus and the CIA group were injected with saline. The third grouping was: Wild, *Esr2*^fl/f^, and *Esr2*^fl/fl^/*Adgre*-Cre. These mice were sacrificed on day 42. Mice were anesthetized by intraperitoneal injection of sodium pentobarbital and then dislocated to death. Wild mice were induced with CIA but without any treatment. *Esr2*^fl/f^ mice and *Esr2*^fl/fl^/*Adgre*-Cre mice were induced with CIA and treated with PSTPIP2 by injection of adeno-associated virus-loaded overexpression *Pstpip2* gene plasmid into the knee joint cavity on day 22.

Synovial tissues from the second and third batches of mice were isolated and then subjected to cell culture. Briefly, the synovial tissues were cut into tiny tissue pieces and then inoculated into culture flasks. After the cells were plastered, it was continued for 24 h, and then F4/80^+^ cells were further cultured using flow sorting for staining and cell dynamics analysis.

Culture of bone marrow-derived mononuclear cells (BMDMs): Bone marrow cells were obtained by aseptically isolating the femur and tibia of DBA/1 mice. After overnight culture (> 16 h), the non-adherent cells were discarded, and the obtained adherent monocytes were continued to be cultured in a medium containing macrophage-colony stimulating factor (M-CSF), to differentiate into macrophages (M0). M0 polarized into M1-type macrophages (F4/80^+^CD86^+^) upon stimulation by IFN-γ and LPS. Under the stimulation of IL-4, M0 polarized into M2-type macrophages (F4/80^+^CD206^+^). AZD9496 (MedChemExpress, Cat. HY-12870) was added to the medium to antagonize estrogen receptor alpha (ERα), Prinaberel (MedChemExpress, Cat. HY-14933) was added to antagonize estrogen receptor beta (ERβ), and Estriol (MedChemExpress, Cat. HY-B0412) was added to antagonize the G protein-coupled estrogen receptor (GPER).

### Flow cytometry and flow sorting

Human peripheral blood and synovial tissue or mouse synovial tissues were used to make single cell suspensions. According to the operation manual, antibodies were added to the treated single cell suspension (1×10^6^/100 μL) and incubated for 30 min at 4°C protected from light. Centrifuged at 300g for 5 min, resuspended by adding 0.5ml of PBS and finally detected on the machine. Directly labeled antibodies, Hu CD11b APC (Cat. # 553312), Hu CD86 FITC (Cat. # 560958), and Hu CD206 PE (Cat. # 555954), were purchased from BD Pharmingen (America). Ms F4/80 PE (Cat. # 565410), Ms CD86 FITC (Cat. # 561962), and Ms CD206 Alexa (Cat. # 565250) were purchased from BD Pharmingen (America). Indirectly labeled antibodies, Anti-PSTPIP2 antibody (Cat. # ab155543), Anti-Claudin5 antibody (Cat. # ab131259), and Anti-ZO-1 antibody (Cat. # ab190085), were purchased from abcam (UK). Fluorescent secondary antibody: Goat Anti-Mouse IgG H&L Alexa (Cat. # ab150113) or Goat Anti-Mouse IgG H&L FITC (Cat. # ab6758) were purchased from abcam (UK).

Single-cell suspensions of human peripheral blood and synovial tissues or mouse synovial tissues were used in part for flow sorting. Manual sorting reagents: Anti-F4/80 MicroBeads UltraPure, mouse (Miltenyi, Cat. # 130-110-443); MiniMACS Starting Kit (Miltenyi, Cat. # 130-090-312); MS Columns (Miltenyi, Cat. # 130-042-201).

### Histology

Hematoxylin-eosin (H&E) staining. The isolated synovial tissues were immediately placed in liquid nitrogen for storage. Frozen tissues were then used for H&E staining, SafraninO-fast green staining, TRAP staining, immunohistochemical staining, and immunofluorescence staining. The antibody used for immunohistochemistry was anti-PSTPIP2 antibody (Cat. # ab155543). The antibodies used for immunofluorescence were anti-CD11b antibody (Cat. # ab52478), anti-PSTPIP2 antibody (Cat. # ab155543), anti-F4/80 antibody (Cat. # ab300421) purchased from abcam (United Kingdom). Isotype control antibodies (Rabbit IgG, monoclonal isotype control, abcam, Cat. # ab172730) were used to eliminate non-specific binding of antibodies in immunohistochemistry and immunofluorescence.

### RT-qPCR

F4/80^+^ synovial macrophages or CD11b^+^ peripheral blood mononuclear cells (isolated using flow sorting) were used to extract total RNA. Total RNA was used for reverse transcription, and finally, fluorescence quantification was performed. CT values were obtained for each sample after the onboard operation and repeated three times. GAPDH was used as an internal standard. GAPDH primer information: Forward 5′-AATGGATTTGGACGCATTGGT-3′, Reverse 5′-TTTGCACTGGTACGTGTTGAT-3′. PSTPIP2 primer information: Forward 5′-ACAACGTGGCTCAATGTCACA-3′, Reverse 5′-CCGGCACTTTTGCTCATAATTCT-3′. The CT values obtained were transformed using the method ΔΔCT. Shortly, A = CT(target gene, sample to be tested) – CT(internal standard gene, sample to be tested), B = CT(target gene, control sample) – CT(internal standard gene, control sample), K = A – B, relative expression of target gene = 2^-K^.

### Micro-CT analysis

The knee joints were separated from the hip joint and removed as much muscle tissue as possible [9]. The isolated ankle and knee were fixed with 4% paraformaldehyde for 24 h. Machine parameters (model: Bruker SkyScan 1276, Belgium): voltage 50 kV, current 800 μA, scan resolution 12 μm, and a field of view size 1304×1024. Several consecutive slices with a large cross-sectional area were used for 3D image reconstruction (N-Recon software) and 3D analysis was performed with CT-AN software. The bone volume fraction (BV/TV, %) was used to indicate the degree of bone damage.

### Construction of adeno-associated virus-loaded overexpression PSTPIP2 gene plasmid

We commissioned Hanheng Biotechnology Ltd. (Shanghai, China) to construct AAV-PSTPIP2 for in vivo overexpression of the *Pstpip2* gene [12, 13]. We finally obtained overexpressed adeno-associated virus (AAV) plasmid (AAV-PSTPIP2) and control (AAV-empty). This project used AAV5, which has a high synovial affinity with infection efficiency of >80% and an RT-qPCR detection level of >3× for PSTPIP2.

### Time-lapse video microscopy

Isolated synovial macrophages were plated on MatTek cell culture dishes at low density and cultured for 2 days before the experiment. Cells were then stained using Actin (Mouse Anti-Actin antibody, abcam, Cat. # ab11003) and PSTPIP2 (Rabbit Anti-PSTPIP2 antibody, abcam, Cat. # ab155543), and secondary antibodies were used as Goat Anti-Mouse IgG(H+L) (FITC conjugated) (Elabscience, Cat. # E-AB-1015) and Goat Anti-Rabbit IgG (H+L) (AF647 conjugated) (Elabscience, Cat. # E-AB-1075). And then the plates were placed on the stage of an Olympus inverted microscope equipped with a humidified, 5% CO2-containing, 37°C temperature-controlled chamber. Phase-contrast images of five different fields were acquired at 60-s intervals using a Cooke Sensicam QE cooled CCD camera (Auburn Hills, MI) with Scanalytics IPLab software (Fairfax, VA) running on a PC. Sequences were assembled and quantified using ImageJ 1.8.0 software. Filopodia formation was assessed by visual inspection of the time-lapse movies [14].

### Scanning electron microscopy

Isolated synovial macrophages were grown on 22-mm circular glass coverslips in six-well cell culture plates to 60–75% confluence. And then the cells were fixed quickly with osmium tetroxide followed by staining with gold-palladium as described [15]. The presence of filopodia and membrane ruffling was examined using a JEOL JSM6400 scanning electron microscope using an accelerating voltage of 5kV. Cell spreading was estimated by manually tracing the cell perimeter and measuring the cell footprint area. Ruffling was estimated by manually tracing the perimeter of the ruffles and measuring the area. The percent ruffling for each cell was obtained by dividing the ruffling area by the sum of the ruffling area and cell area. The number and length of filamentous protrusions were determined by counting and manual tracing, respectively [14].

### Laser confocal microscopy

Laser confocal microscopy is used to photograph cells or tissues with fluorescence. Anti-fluorescent bursting agents are added dropwise to the prepared cell crawl or tissue section and photographed on the machine [16]. Olympus laser confocal microscope (FV3000) was used in this study.

### Statistical analyses

All data are expressed as mean ± SD. Unpaired *t*-test was used for two groups, and one-way ANOVA test was used for three groups or more. All data met the assumption of statistical tests. The number of mice in each group is illustrated in the figure. The grouping of mice was randomized. Histological scoring was performed in a blinded fashion. Results were considered significant at *P* < 0.05. Statistical tests were carried out using GraphPad Prism (version 8.01) Software for Windows.

## Results

### The level of PSTPIP2 in synovial macrophages is negatively correlated with RA disease activity, which is mediated by synovial macrophages polarization

**Fig. 1:**
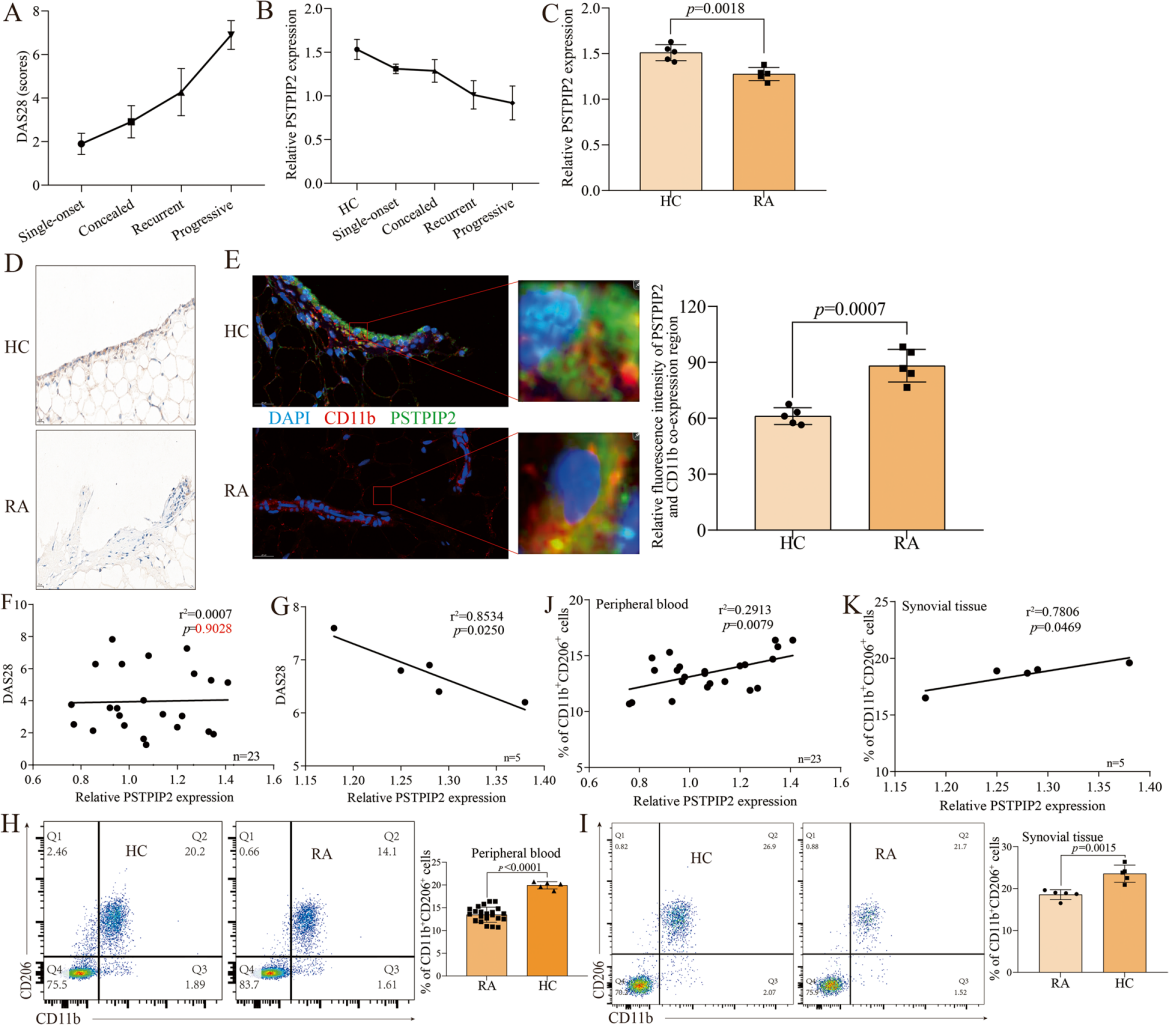
The level of PSTPIP2 in synovial macrophages is negatively correlated with RA disease activity, which was mediated by synovial macrophages polarization. **A** DAS28 score of 23 RA patients, including 5 single-onset type, 6 concealed type, 6 recurrent type, and 6 persistently progressive types. **B** The expression of PSTPIP2 in peripheral blood CD11b^+^ monocytes of 5 healthy control and 23 RA patients. **C** The expression of PSTPIP2 in CD11b^+^ synovial macrophages of 5 healthy control and 5 persistently progressive RA patients. **D** Immunohistochemical staining of synovial tissues in 5 healthy control and 5 persistently progressive RA patients. **E** Immunofluorescence double staining of synovial tissues in 5 healthy control and 5 persistently progressive RA patients. **F** Correlation analysis of PSTPIP2 level in peripheral blood CD11b^+^ monocytes with DAS28 in 23 RA patients. **G** Correlation analysis of PSTPIP2 level in CD11b^+^ synovial macrophages with DAS28 in 5 persistently progressive RA patients. **H** Scatter plot (left) and histogram (right) of CD11b^+^CD206^+^ cells in peripheral blood. **I** Scatter plot (left) and histogram (right) of CD11b^+^CD206^+^ cells in synovial tissue. **J** Correlation analysis of the level of PSTPIP2 in CD11b^+^ monocytes with CD11b^+^CD206^+^ cells in peripheral blood. **K** Correlation analysis of the level of PSTPIP2 in CD11b^+^ monocytes with CD11b^+^CD206^+^ cells in synovial tissue. *P* < 0.05 indicates that the difference is statistically significant. Data represent mean ± SD (unpaired *t* test for **D**, **H**, and **I**; linear regression for **F**, **G**, **J**, and **K**)

Our previous animal studies revealed that overexpression of PSTPIP2 in the joint cavity improved articular bone damage in arthritic rats. To further elucidate the role of PSTPIP2 in the clinical setting, clinical samples were used to explore. 23 RA patients (experimental group) and five acute trauma patients (healthy controls) were enrolled in the study with the assistance of the orthopedic department and rheumatology department. The 23 RA patients included 5 single-onset cases, 6 concealed cases, 6 recurrent cases, and 6 persistently progressive cases. Clinical data and peripheral blood from 23 RA patients and 5 acute trauma patients, synovial tissues from 5 progressive RA patients, and 5 acute trauma patients were obtained by screening and inclusion and exclusion criteria. Blood parameters showed an increasing trend of rheumatoid factor (RF) (Supl. Fig. 1A), antistreptolysin (ASO) (Supl. Fig. 1B), and DAS28 score (Fig. 1A) with increasing RA disease activity. Next, We isolated peripheral blood CD11b^+^ monocytes using flow sorting, and RT-qPCR analysis indicated that the expression of PSTPIP2 in CD11b^+^ monocytes tended to decrease progressively with increasing RA disease activity (Fig. 1B). Further, PSTPIP2 level was significantly decreased in CD11b^+^ synovial macrophages from progressive RA patients (Fig. 1C). Immunohistochemistry of synovial tissues corroborated these findings, as PSTPIP2 levels were down-regulated in RA patients and were mainly expressed in synovial macrophages (Fig. 1D). Immunofluorescence double staining of CD11b and PSTPIP2 further confirmed the high expression of PSTPIP2 in synovial macrophages, and PSTPIP2 expression was decreased in RA (Fig. 1E). Interestingly, linear regression analysis showed that PSTPIP2 level in peripheral blood CD11b^+^ monocytes did not correlate with the DAS28 scores (Fig. 1F), whereas PSTPIP2 level in synovial tissues CD11b^+^ macrophages correlated negatively with the DAS28 scores (Fig. 1G). Importantly, PSTPIP2 level in CD11b^+^ monocytes correlated positively with the frequency of CD11b^+^CD206^+^ cells (Fig. 1H and J) but not with the frequency of CD11b^+^CD86^+^ cells (Supl. Fig. 1C and E) in the peripheral blood of RA patients. Consistently, the same phenomenon was observed in synovial macrophages (Fig. 1I and K) (Supl. Fig. 1D and F). These data suggest that the level of PSTPIP2 in synovial macrophages is negatively correlated with RA disease activity, which is mediated by synovial macrophages polarization.

### PSTPIP2^hi^ synovial macrophages form a tight immunological barrier in the lining layer

**Fig. 2:**
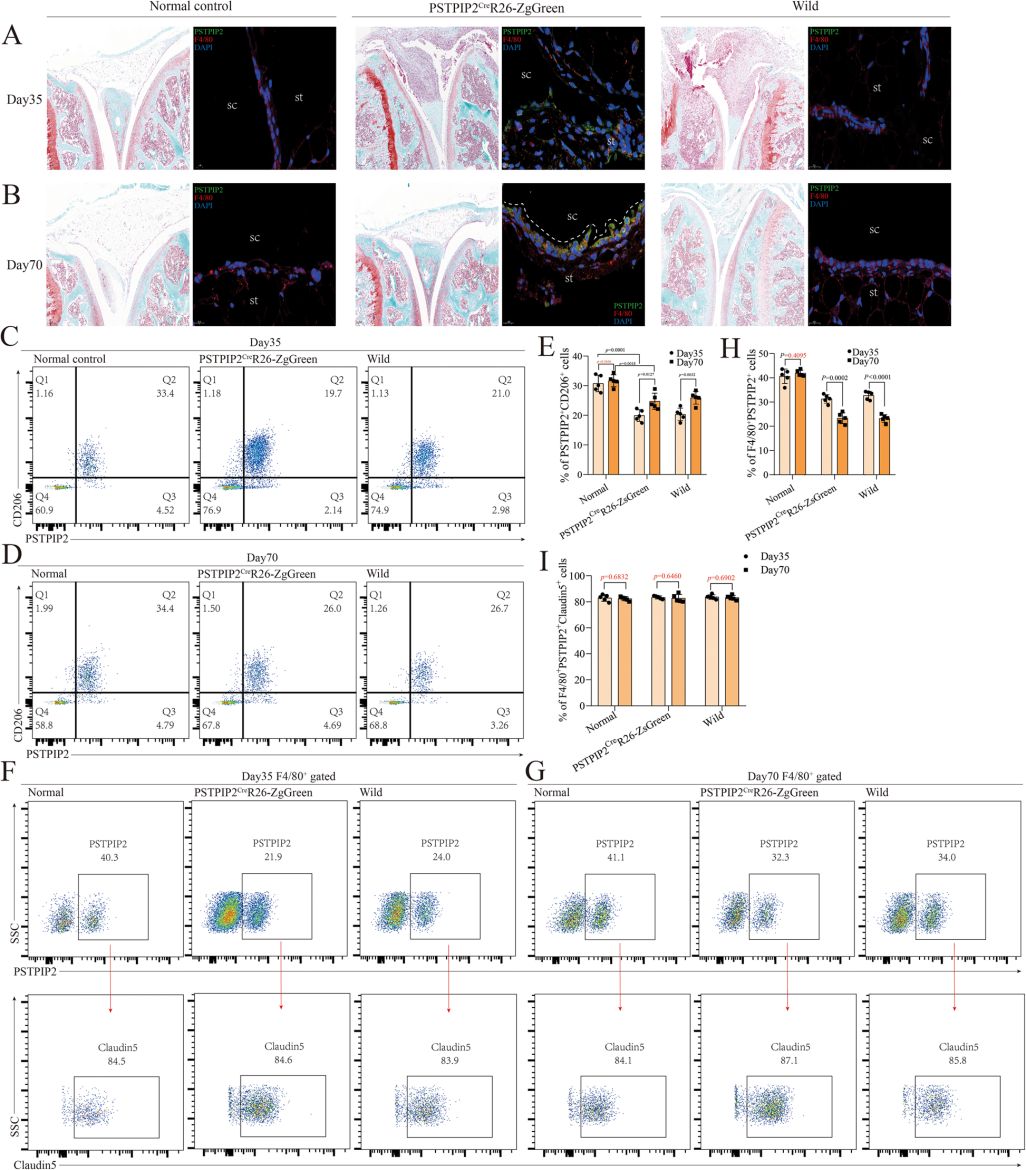
PSTPIP2^hi^ synovial macrophages form a tight immunological barrier in the lining layer. **A** SafraninO-fast green staining and F4/80 fluorescence staining of synovial tissue in normal controls, *Pstpip2*^Cre^R26-*ZsGreen* reporter mice, and wild mice on day 35. PSTPIP2 (green), F4/80 (red), and DAPI (blue); sc, synovial cavity; st, synovial tissue. **B** SafraninO-fast green staining and CD206 fluorescence staining of synovial tissue in normal controls, *Pstpip2*^Cre^R26-*ZsGreen* reporter mice, and wild mice on day 70. **C** Scatter plot of PSTPIP2^hi^CD206^+^ synovial macrophages in synovial tissue on day 35. **D** Scatter plot of PSTPIP2^hi^CD206^+^ synovial macrophages in synovial tissue on day 70. **E** Histogram of PSTPIP2^hi^CD206^+^ synovial macrophages in synovial tissue on day 35 and day 70. **F** Scatter plot of claudin5 expression in F4/80^+^PSTPIP2^+^ synovial macrophages on day 35. **G** Scatter plot of claudin5 expression in F4/80^+^PSTPIP2^+^ synovial macrophages on day 70. **H** Histogram of F4/80^+^PSTPIP2^+^ synovial macrophages in synovial tissue on day 35 and day 70. **I** Histogram of F4/80^+^PSTPIP2^+^claudin5^+^ synovial macrophages in synovial tissue on day 35 and day 70. *P* < 0.05 indicates that the difference is statistically significant. Data represent mean ± SD (unpaired *t* test for **E**, **H**, and **I**)

To investigate the role of PSTPIP2 in bone erosion, we introduced *Pstpip2*^Cre^R26-*ZsGreen* mice to trap the PTPIP2 protein. After mating, breeding, and screening, 10 female *Pstpip2*^Cre^R26-*ZsGreen* mice (observation group), 10 wild-type mice (control group), and 10 normal control mice were eventually used to establish the collagen-induced arthritis (CIA) model. The joint inflammation peaked on day 35 and began to remit on day 56. Each group of 10 mice was further randomly divided into 2 groups, the peak group (day 35) and the remission group (day 70), to observe the local distribution of PSTPIP2 protein in the joints during the progressive and remission phases of CIA. PSTPIP2-positive cells were predominantly enriched in the synovial lining (Fig. 2A, B), and synovial macrophages highly expressing PSTPIP2 (PSTPIP2^hi^) form a barrier in the lining layer (Fig. 2A, B). During the peak of inflammation in CIA, the barrier is thin and not compact, whereas during CIA remission the barrier becomes thick and compact (Fig. 2A, B). Consistently, flow cytometry results also demonstrated that the proportion of PSTPIP2^hi^CD206^+^ cells was elevated in remission compared to peak (Fig. 2C–E). Not only that, the percentage of PSTPIP2^hi^CD206^+^ cells in the joint was significantly lower compared to normal controls, both at peak and in remission (Fig. 2C–E). In normal mice, about 40% of synovial macrophages expressed PSTPIP2, compared to only about 20% in CIA mice (Fig. 2F–I). Notably, about 85% of PSTPIP2^+^ synovial macrophages expressed Claudin5 (tight junction protein) (Fig. 2F–I) and zonula occludens-1 (ZO-1) (Supl. Fig. 2A, B). These data suggest that PSTPIP2 is highly expressed in macrophages in the synovial lining and that PSTPIP2^hi^ synovial macrophages form a tight immunological barrier in the lining.

### PSTPIP2 regulates synovial macrophages polarization in vivo

**Fig. 3:**
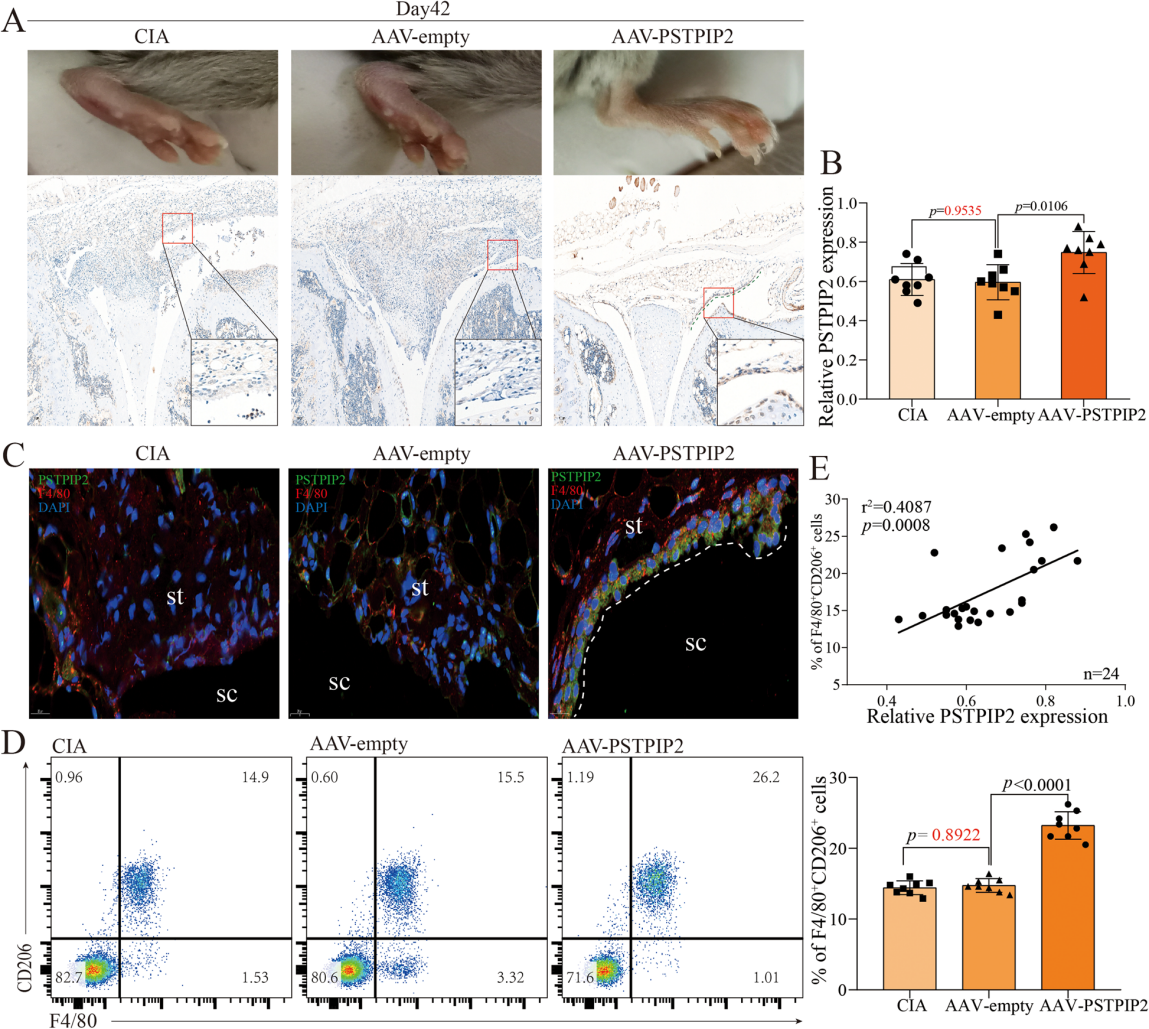
PSTPIP2 regulates synovial macrophages polarization in vivo. **A** Photographs of mouse paws and immunohistochemistry of the knee joint (brown: PSTPIP2). CIA: collagen-induced arthritis group. AAV-empty: control group. AAV-PSTPIP2: Adeno-associated virus loaded with PSTPIP2 plasmid group. **B** Expression of PSTPIP2 in F4/80^+^ synovial macrophages. F4/80^+^ macrophages in synovial tissue were isolated using flow sorting and then PSTPIP2 expression was detected by RT-qPCR. **C** Immunofluorescence double staining of synovial tissues. Green: PSTPIP2, red: F4/80. sc, synovial cavity; st, synovial tissue. **D** Analysis of synovial macrophages polarization: the percentage of F4/80^+^CD206^+^ cells in synovial tissue. Scatter plot on the left, histogram on the right. **E** Correlation analysis of PSTPIP2 level in F4/80^+^ synovial macrophages with the frequency of F4/80^+^CD206^+^ cells in synovial tissue. *P* < 0.05 indicates that the difference is statistically significant. Data represent mean ± SD (one-way ANOVA for **B** and **D**; linear regression for **E**)

To further clarify the role of PSTPIP2 in RA and its molecular mechanisms, the CIA model of the DBA/1 mouse was used for follow-up studies. The course of CIA was: peak inflammation from day 35 to day 56, with remission starting on day 56 (Supl. Fig. 3A and B). On day 35, the knee and ankle joints exhibited synovial hyperplasia, inflammatory cell infiltration, and pannus formation, while these inflammations improved on day 70 (Supl. Fig. 3C). Importantly, PSTPIP2 level in synovial macrophages was negatively correlated with the severity of CIA (Supl. Fig. 3D-F). Further, PSTPIP2 was locally overexpressed in the knee joint cavity to observe the therapeutic effect of PSTPIP2 on CIA. PSTPIP2 was successfully overexpressed in F4/80^+^ synovial macrophages (Fig. 3A and B), and PSTPIP2 improved joint inflammation and bone damage in CIA mice (Supl. Fig. 4A and B). PSTPIP2 protein increased the frequency of F4/80^+^CD206^+^ synovial macrophages (Fig. 3D) and decreased the frequency of F4/80^+^CD86^+^ synovial macrophages (Supl. Fig. 4C). Consistent with the human data, PSTPIP2 level in F4/80^+^ synovial macrophages were positively correlated with the frequency of F4/80^+^CD206^+^ synovial macrophages (Fig. 3E), whereas there was no correlation with the frequency of F4/80^+^CD206^+^ synovial macrophages (Supl. Fig. 4D). Notably, the PSTPIP2^hi^ synovial macrophages barrier was observed in the overexpression group, but not in the control and CIA groups ((Fig. 3C). These evidence reveal that PSTPIP2 regulates synovial macrophages polarization, and the anti-inflammatory effect of PSTPIP2 protein is synergistic with that of F4/80^+^CD206^+^ synovial macrophages. PSTPIP2^hi^ cells form an immunological barrier in the synovial lining layer to resist inflammatory erosion of the joint.

### The ability of PSTPIP2 to regulate synovial macrophages polarization is dependent on ERβ

**Fig. 4:**
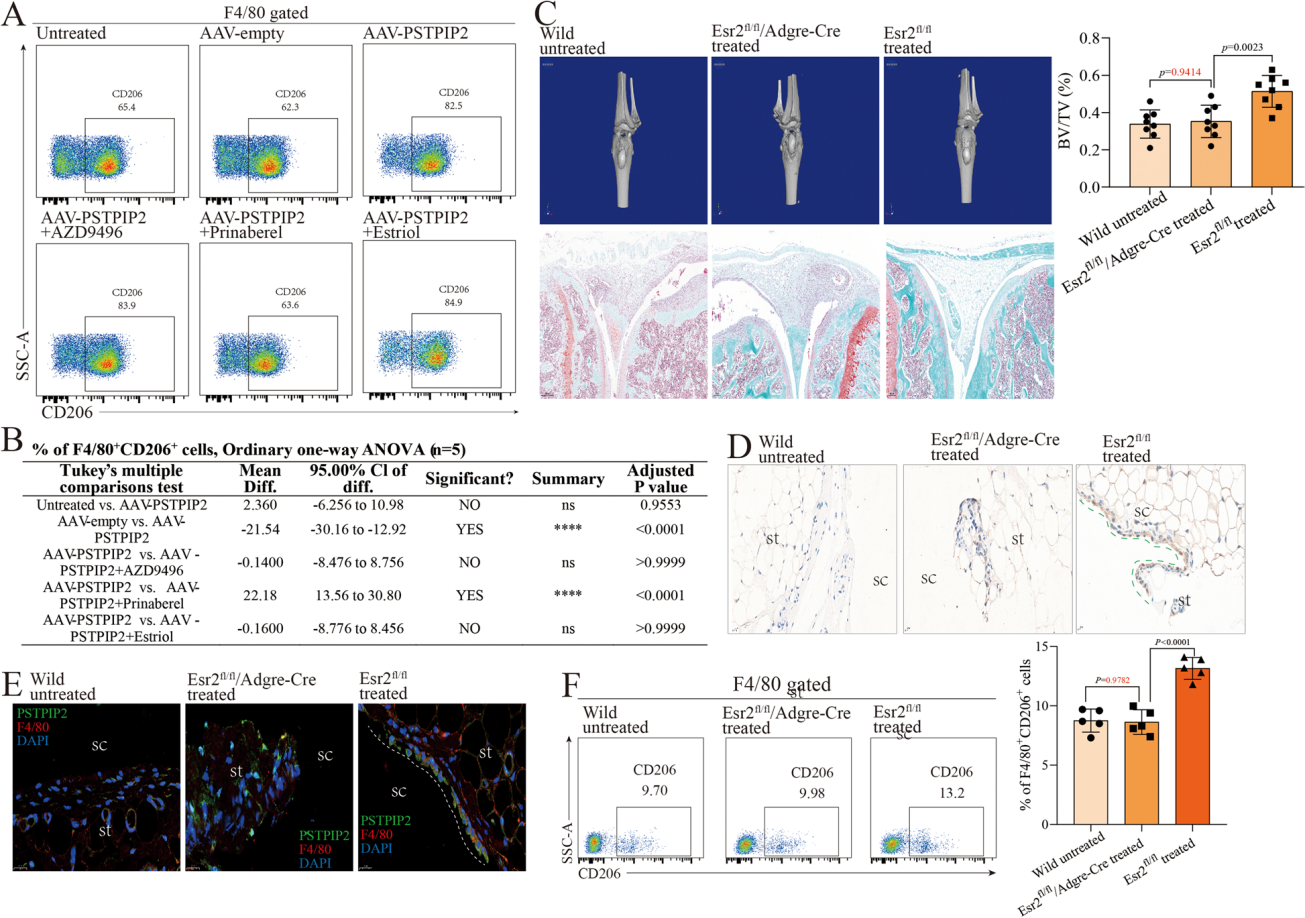
The ability of PSTPIP2 to regulate synovial macrophages polarization is dependent on ERβ. **A** Effect of estrogen receptors on macrophage polarization in vitro: Scatter plot of F4/80^+^CD206^+^ cells. AZD9496: selective ERα antagonist, Prinaberel: selective ERβ antagonist, Estriol: G protein-coupled estrogen receptor antagonist. **B** One-way ANOVA data for F4/80^+^CD206^+^ cells. **C** micro-CT and SafraninO-fast green staining of knee joint. The bar chart on the right shows BV/TV analyzed from micro-CT data. BV/TV: bone volume/total volume. **D** immunohistochemistry of the knee joint (brown: PSTPIP2). sc: synovial cavity, st: synovial tissue. **E** Immunofluorescence double staining of synovial tissue for PSTPIP2 and F4/80. Green: PSTPIP2, red: F4/80, blue: DAPI. **F** Analysis of synovial macrophages polarization: the percentage of F4/80^+^CD206^+^ cells in synovial tissue. Scatter plot on the left, histogram on the right. *P* < 0.05 indicates that the difference is statistically significant. Data represent mean ± SD (one-way ANOVA for **B**, **C**, and **F**)

Our studies have shown that local expression of PSTPIP2 regulates the polarization of synovial macrophages, however, the molecular mechanism by which PSTPIP2 regulates polarization is uncertain. To this end, our laboratory has done extensive work, including proteomics, gene sequencing, and bioinformatics analysis. After preliminary screening, we found that the anti-bone damage ability of PSTPIP2 may be associated with the estrogen receptor. In vitro cultured bone marrow-derived monocytes (BMDMs) were used to probe the relationship between PSTPIP2 protein and the estrogen receptor in macrophages polarization. Briefly, BMDMs were stimulated to become macrophages (M0), which are further stimulated to polarize to M1 or M2. In the process of macrophages polarization, we interfered with PSTPIP2 expression by transfecting adeno-associated viruses loaded with plasmids overexpressing the *Pstpip2* gene and with the estrogen receptor by using estrogen receptor antagonists. Interestingly, blocking ERα and the G protein-coupled estrogen receptor (GPER) did not affect PSTPIP2-mediated macrophages polarization (Supl. Fig. 5A and B), whereas the ability of PSTPIP2 to regulate macrophages polarization was lost only when ERβ was blocked (Fig. 4A and B). To investigate this phenomenon in depth, wild-type mice, *Esr2*^fl/fl^ mice (control), and *Esr2*^fl/fl^/*Adgre*-Cre mice (F4/80^+^ macrophages-specific knockout of ERβ) were introduced for animal experiments. All mice were induced with CIA and PSTPIP2 was overexpressed in the knee joint cavity of *Esr2*^fl/fl^ mice *Esr2*^fl/fl^/*Adgre*-Cre mice. The results showed that the anti-erosion effect of PSTPIP2 was lost in *Esr2*^fl/fl^/*Adgre*-Cre mice (Fig. 4C). *Esr2*^fl/fl^ mouse synovial tissue was enriched with PSTPIP2 protein in the synovial lining layer and PSTPIP2-positive cells were neatly arranged (Fig. 4D). However, PSTPIP2 protein in synovial tissue from *Esr2*^fl/fl^/*Adgre*-Cre mice was scattered and largely absent in wild type (Fig. 4D). Consistently, PSTPIP2^hi^ synovial macrophages in synovial tissue of *Esr2*^fl/fl^ mice formed a barrier in the lining layer, whereas PSTPIP protein distribution in synovial tissue of *Esr2*^fl/fl^/*Adgre*-Cre mice was scattered and CD206 level was low (Fig. 4E). Further, the ability of PSTPIP2 to regulate the polarization of synovial macrophages was lost in *Esr2*^fl/fl^/*Adgre*-Cre mice (Fig. 4F) (Supl. Fig. 5C). These evidence reveals that the ability of PSTPIP2 to regulate synovial macrophages polarization to resist bone erosion is dependent on ERβ.

### PSTPIP2 regulates the dynamics of synovial macrophages via ERβ

**Fig. 5:**
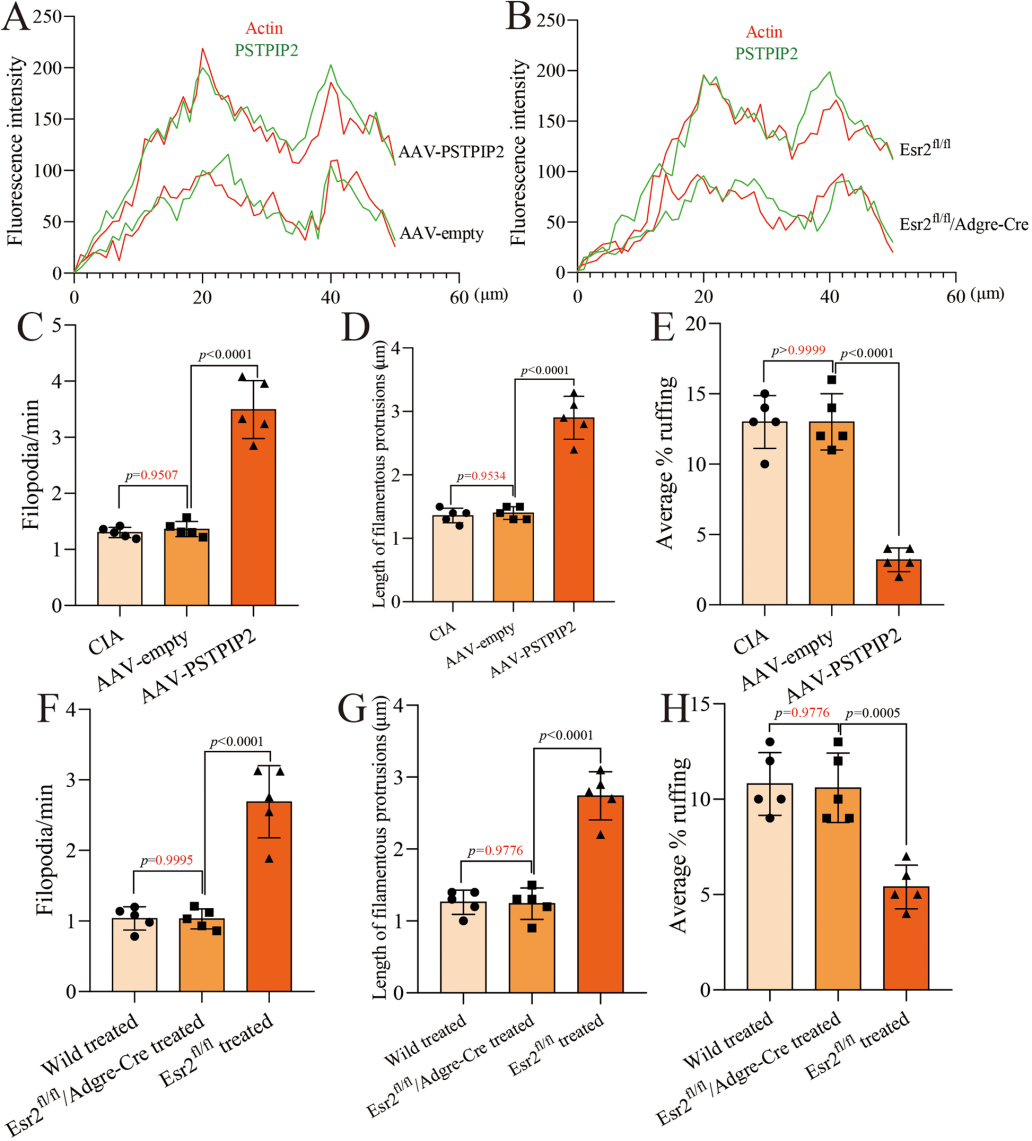
PSTPIP2 regulates the dynamics of synovial macrophages, which is associated with ERβ. **A**, **B** Synovial macrophages derived from the mouse synovial tissue (including AAV-empty group, AAV-PSTPIP2 group, *Pstpip2*^Cre^R26-*ZsGreen* mice, and Esr2^fl/fl^ mice) were fixed and stained with antibody against Actin (red) or PSTPIP2 (green). Fiji software was used to analyze the fluorescence intensity. **C**, **F** Dynamics of filopodia formation in synovial macrophages. The rate of filopodia formation in living cells was determined from time-lapse movies by counting the number of new filopodia formed in each frame. **D**, **G** Quantitative analysis of the length of filamentous protrusions. **F**, **H** Quantitative analysis of the membrane ruffling. *P* < 0.05 indicates that the difference is statistically significant. Data represent mean ± SD (one-way ANOVA for **C**–**H**)

Synovial macrophages with high expression of PSTPIP2 formed an immunological barrier in the lining layer. We suggest that PSTPIP2, F-Bar protein, may affect the migration of synovial macrophages. Synovial macrophages were isolated from synovial tissue of experimental mice for culture. Upon staining and microscopic observation, we found that PSTPIP2 and Actin were at synchronous levels in synovial macrophages and that PSTPIP2 overexpressing synovial macrophages also exhibited high levels of Actin (Fig. 5A and B). Interestingly, the ability of PSTPIP2 to elevate Actin was not observed in *Esr2*^fl/fl^/*Adgre*-Cre mice (Fig. 5B). Further, PSTPIP2 promoted the formation of filopodia in synovial macrophages and inhibited the formation of membrane ruffing (Fig. 5C-E). Overexpression of PSTPIP2 in the knee of *Esr2*^fl/fl^/*Adgre*-Cre mice failed to regulate the formation of filopodia and membrane ruffing in synovial macrophages compared to *Esr2*^fl/fl^ mice (Fig. 5F–H). These evidence indicate that PSTPIP2 is able to regulate the dynamics of synovial macrophages, which is dependent on ERβ.

## Discussion

The current global prevalence of RA is approximately 1% and severe RA patients may lose mobility due to joint damage. In RA, PSTPIP2 is mainly locally overexpressed in synovial macrophages and PSTPIP2^hi^ synovial macrophages form an immunological barrier in the synovial lining layer to protect the joint from erosion. Therefore, local overexpression of PSTPIP2 in the joint cavity may be a potential strategy for the treatment of bone damage in RA patients.

PSTPIP2 is a member of the F-Bar protein family and plays an important role in auto-inflammatory diseases [17]. Previous studies have demonstrated the anti-inflammatory and anti-bone erosion effects of PSTPIP2 [9, 18]. However, the role of PSTPIP2 in immunity is still a mystery. The present study reveals that the anti-bone erosion ability of PSTPIP2 is mediated by synovial macrophages polarization. Interestingly, the anti-bone erosion effect of PSTPIP2 may be local rather than systemic. Because PSTPIP2 level in peripheral blood CD11b^+^ monocytes did not correlate with RA disease activity (DAS28), whereas PSTPIP2 level in CD11b^+^ macrophages in synovial tissue was negatively correlated with RA disease activity.

Furthermore, PSTPIP2 was able to promote the polarization of synovial macrophages into F4/80^+^CD206^+^ cells. This suggests that PSTPIP2 exerts its anti-bone erosion effect through local regulation of synovial macrophages polarization. Data from human samples and animal studies revealed that PSTPIP2 level in synovial macrophages was positively correlated with CD206^+^ cells in synovial tissue, but not with CD86^+^ cells. This is an interesting phenomenon, which suggests a possible mechanism by which PSTPIP2 regulates the polarization of synovial macrophages: PSTPIP2 directly promotes the differentiation of synovial macrophages towards CD206^+^ cells, while indirectly influencing the generation of CD86^+^ cells. Stefano Alivernini et al. found that different subtypes of synovial macrophages have different abilities to regulate homeostasis and inflammation [19]. As a complement, PSTPIP2^+^CD206^+^ synovial macrophage plays crucial in the joint microenvironment. Whether PSTPIP2^+^CD206^+^ synovial macrophage is a new subgroup still needs more data to support, such as single-cell RNA sequencing (scRNA-seq). A study published in *Nature* showed that locally renewed synovial macrophages can provide a protective barrier for the joints [20]. Differently, our study showed that synovial macrophages with high PSTPIP2 expression tended to polarize to the M2 type. And PSTPIP2^hi^CD206^+^ synovial macrophages tend to move toward the synovial lining layer and form an anti-inflammatory barrier.

The application of *Pstpip2*^Cre^R26-*ZsGreen* reporter mice confirms our findings from the studies with clinical samples. *Pstpip2*^Cre^R26-*ZsGreen* reporter mice were generated by gene editing and were able to show the location of PSTPIP2 protein (green) in vivo. During the peak inflammatory phase of CIA, PSTPIP2 level was low in synovial tissues, while during remission it was significantly elevated. Furthermore, synovial macrophages with high expression of PSTPIP2 (PSTPIP2^hi^) were enriched in the synovial lining and PSTPIP2^hi^ synovial macrophages formed an immunological barrier (F4/80^+^PSTPIP2^hi^ cell enriched area) in the lining. The distribution of PSTPIP2 in the knee joint cavity was scattered and the level was low during the peak inflammatory phase of arthritis, but the distribution became concentrated and the level became high during remission. Thus, the barrier formed by PSTPIP2^hi^ synovial macrophages changes dynamically with the development of inflammation. However, in the present study, we selected only 2 observation sites due to experimental constraints, which are limited. More observation sites are needed to more clearly observe the role of dynamic changes in the PSTPIP2^hi^ cell barrier in CIA joint bone erosion.

Interestingly, synovial macrophages exhibit a high degree of heterogeneity in RA. The persistence of synovial tissue macrophages during RA remission contributes to joint homeostasis [19]. By scRNA-seq of RA synovial tissue, Stefano Alivernini et al. identified two subpopulations of synovial macrophages (MerTK^pos^TREM2^high^ and MerTK^pos^LYVE1^pos^) with a unique remitting transcriptome profile, enriched for negative regulators of inflammation [19]. The study found that MerTK^neg^CD206^neg^ and MerTK^pos^CD206^pos^ synovial macrophage populations have distinct pro- and anti-inflammatory phenotypes [19]. Based on these data, and combined with the present study, it is suggested that PSTPIP2^hi^ may belong to a unified cell cluster with MerTK^pos^. However, the limitations of the present study (e.g., the use of mouse synovial tissue in this study) do not allow for elucidation of this point. Therefore, further scRNA-seq is necessary to elucidate the unique isoform of PSTPIP2^hi^ synovial macrophages. Regardless, either PSTPIP2^hi^ or MerTK^pos^ synovial macrophages exhibit excellent anti-inflammatory properties in the joint microenvironment. Taking advantage of the aggregated anti-inflammatory function of PSTPIP2^hi^ synovial macrophages is important to alleviate inflammation in the joint microenvironment.

Previous studies have shown that mutation in *PSTPIP2* causes macrophage-associated inflammatory diseases [7]. And methylation of *PSTPIP2* is associated with hepatic macrophage polarization in alcoholic liver disease [6, 21]. However, more studies have been reported to reveal the role of PSTPIP2 in inflammatory bone diseases [18, 22, 23]. Consistently, all of these studies focused on macrophages. Similarly, the present study found that the anti-bone erosion capacity of PSTPIP2 was mediated by synovial macrophages. Although the role of PSTPIP2 in regulating macrophages polarization is well established, its molecular mechanisms are unclear. Notably, this study reveals that the anti-erosion capacity of PSTPIP2 is dependent on estrogen receptor β (ERβ) in synovial macrophages and that PSTPIP2 is able to regulate the dynamics of synovial macrophages.

Our laboratory has been working to explore the molecular mechanisms underlying the resistance of PSTPIP2 to bone erosion. To this end, we have done extensive work, including proteomics, gene sequencing, and bioinformatics. After preliminary screening, we found that the ability of PSTPIP2 to regulate the polarization of synovial macrophages is likely to be associated with the estrogen receptor. There are five main types of estrogen receptors: estrogen receptor α (ERα), estrogen receptor β (ERβ), and G protein-coupled estrogen receptor (GPER) are associated with the skeletal system [24–26], and two others, Gaq-ER and ER-X, are mainly associated with the nervous system [27]. To this end, we performed a pilot study on bone marrow-derived monocytes targeting ERα, ERβ, and GPER. Surprisingly, PSTPIP2 was able to regulate macrophages polarization only when ERβ was not antagonized. Further, we introduced macrophages-specific knockout *Esr2* gene (encoding ERβ) in mice (*Esr2*^fl/fl^/*Adgre*-Cre mice) to validate our findings. As expected, in *Esr2*^fl/fl^/*Adgre*-Cre mice, PSTPIP2 completely lost its ability to resist bone erosion and failed to regulate the polarization of synovial macrophages. It is worth noting that we also found PSTPIP2 regulates synovial macrophages dynamics, which is also dependent on ERβ. Overall, ERβ is a key factor in the function of PSTPIP2 in macrophages.

However, there are also limitations of the present study. We did not discuss whether interstitial macrophages could differentiate into lining macrophages. In terms of studying PSTPIP2 and cellular dynamics, we did not study it in depth. We showed changes in actin and filopodia without providing evidence of macrophage migration from the synovial sublayer to the lining layer. In addition, in animal experiments with overexpression of PSPTIP2, we only used immunohistochemistry and RT-qPCR for validation. Validation of the efficiency of in vivo overexpression of PSTPIP2 at the protein level is necessary.

## Conclusions

In conclusion, this study demonstrates that PSTPIP2 protein has a strong anti-bone erosion ability in RA (graphical abstract). The mechanisms are: (i) PSTPIP2 can promote the polarization of synovial macrophages into F4/80^+^CD206^+^ cells to exert anti-inflammatory effects via ERβ. (ii) PSTPIP2 promotes the formation of filopodia and inhibits the formation of membrane ruffing to promote synovial macrophages dynamics via ERβ. (iii) PSTPIP2^hi^ synovial macrophages migrate more readily into the lining layer, forming a tight immune barrier (F4/80^+^ PSTPIP2^hi^ cell-enriched zone) that prevents subsynovial bone erosion. Thus, local modulation of PSTPIP2 expression in the joint cavity may be a potential strategy for controlling bone erosion in rheumatoid arthritis.

## Supplementary Information

**Additional file 1: Table 1.** Clinical information of the participants (mean ± SD). **Supplementary Figure 1.** (A) RF levels in RA patients. (B) ASO levels in RA patients. (C) Scatter plot (left) and histogram (right) of CD11b^+^CD86^+^ cells in peripheral blood. (D) Scatter plot (left) and histogram (right) of CD11b^+^CD86^+^ cells in synovial tissue. (E) Correlation analysis of the level of PSTPIP2 in CD11b^+^ monocytes with CD11b^+^CD86^+^ cells in peripheral blood. (F) Correlation analysis of the level of PSTPIP2 in CD11b^+^ monocytes with CD11b^+^CD86^+^ cells in synovial tissue. *P* < 0.05 indicates that the difference is statistically significant. (G) Peripheral blood monocyte/macrophage gating strategy. (H) Synovial tissue monocyte/macrophage gating strategy. Data represent mean ± SD (unpaired t test for C and D; linear regression for E and F). **Supplementary Figure 2.** (A) Scatter plot of ZO-1 expression in F4/80^+^PSTPIP2^+^ synovial macrophages on day35. (B) Scatter plot of ZO-1 expression in F4/80^+^PSTPIP2^+^ synovial macrophages on day70. (C) Histogram of F4/80^+^PSTPIP2^+^ZO-1^+^ synovial macrophages in synovial tissue on day35 and day70. *P* < 0.05 indicates that the difference is statistically significant. Data represent mean ± SD (unpaired t test for C). **Supplementary Figure 3.** (A) Fold plot of arthritis score over time in CIA mice. (B) Photographs of the paws of mice at different time points. (C) HE staining of the knee in mice at different time points. (D) Expression of PSTPIP2 in F4/80^+^ macrophages at different time points in synovial tissue. (E) Immunofluorescence staining of PSTPI2P in synovial tissues at different time points. Green: PSTPIP2, Blue: DAPI. (F) Correlation analysis of PSTPIP2 levels in F4/80^+^ synovial macrophages with arthritis scores in the CIA model. *P* < 0.05 indicates that the difference is statistically significant. Data represent mean ± SD (one-way ANOVA for D; linear regression for F). **Supplementary Figure 4.** (A) SafraninO-fast green staining of the knee joint. (B) TRAP staining of the knee joint. (C) Analysis of synovial macrophages polarization: the percentage of F4/80^+^CD86^+^ cells in synovial tissue. Scatter plot on the left, histogram on the right. (D) Correlation analysis of PSTPIP2 level in F4/80^+^ synovial macrophages with the frequency of F4/80^+^CD86^+^ cells in synovial tissue. *P* < 0.05 indicates that the difference is statistically significant. Data represent mean ± SD (one-way ANOVA for C; linear regression for D). **Supplementary Figure 5.** (A) Effect of estrogen receptors on macrophage polarization *in vitro*: Scatter plot of F4/80^+^CD86^+^ cells. AZD9496: selective ERα antagonist, Prinaberel: selective ERβ antagonist, Estriol: G protein-coupled estrogen receptor antagonist. (B) Histogram of F4/80^+^CD86^+^ cells (left). One-way ANOVA data for F4/80^+^CD206^+^ cells (right). (C) Analysis of synovial macrophages polarization: the percentage of F4/80^+^CD86^+^ cells in synovial tissue. Scatter plot on the left, histogram on the right. *P* < 0.05 indicates that the difference is statistically significant. Data represent mean ± SD (one-way ANOVA for B and C).

## Data Availability

All data generated or analyzed during this study are included in this published article [and its supplementary information files].

## Abbreviations

AAV: Adeno-associated virus
ASO: Antistreptolysin
BMDMs: Bone marrow-derived monocytes
CIA: Collagen-induced arthritis
ER: Estrogen receptor
GPER: G protein-coupled estrogen receptor
M0: Unpolarized macrophages
M1: Polarized M1-type macrophages
M2: Polarized M2-type macrophages
M-CSF: Macrophage-colony stimulating factor
RA: Rheumatoid arthritis
RF: Rheumatoid factor
ZO-1: Zonula occludens-1

## References

[1] Weyand CM, Goronzy JJ (2021). The immunology of rheumatoid arthritis. Nat Immunol.

[2] Ibanez-Costa A, Perez-Sanchez C, Patino-Trives AM, Luque-Tevar M, Font P, Arias de la Rosa I, et al. Splicing machinery is impaired in rheumatoid arthritis, associated with disease activity and modulated by anti-TNF therapy. Ann Rheum Dis. 2022;81(1):56–67. 10.1136/annrheumdis-2021-220308 PMC8762032 34625402

[3] Smolen JS, Aletaha D, Barton A, Burmester GR, Emery P, Firestein GS, Kavanaugh A, McInnes IB, Solomon DH, Strand V (2018). Rheumatoid arthritis. Nat Rev Dis Primers.

[4] Firestein GS, McInnes IB (2017). Immunopathogenesis of Rheumatoid Arthritis. Immunity.

[5] Drobek A, Kralova J, Skopcova T, Kucova M, Novak P, Angelisova P, Otahal P, Alberich-Jorda M, Brdicka T (2015). PSTPIP2, a Protein Associated with Autoinflammatory Disease, Interacts with Inhibitory Enzymes SHIP1 and Csk. J Immunol.

[6] Yang Y, Wu XQ, Li WX, Huang HM, Li HD, Pan XY, Li XF, Huang C, Meng XM, Zhang L (2018). PSTPIP2 connects DNA methylation to macrophage polarization in CCL4-induced mouse model of hepatic fibrosis. Oncogene.

[7] Grosse J, Chitu V, Marquardt A, Hanke P, Schmittwolf C, Zeitlmann L, Schropp P, Barth B, Yu P, Paffenholz R (2006). Mutation of mouse Mayp/Pstpip2 causes a macrophage autoinflammatory disease. Blood.

[8] Chitu V, Ferguson PJ, de Bruijn R, Schlueter AJ, Ochoa LA, Waldschmidt TJ, Yeung YG, Stanley ER (2009). Primed innate immunity leads to autoinflammatory disease in PSTPIP2-deficient cmo mice. Blood.

[9] Yao Y, Cai X, Yu H, Xu Q, Li X, Yang Y, Meng X, Huang C, Li J (2019). PSTPIP2 attenuates joint damage and suppresses inflammation in adjuvant-induced arthritis. Eur J Pharmacol.

[10] Aletaha D, Neogi T, Silman AJ, Funovits J, Felson DT, Bingham CO, Birnbaum NS, Burmester GR, Bykerk VP, Cohen MD (2010). 2010 rheumatoid arthritis classification criteria: an American College of Rheumatology/European League Against Rheumatism collaborative initiative. Ann Rheum Dis.

[11] Hu XX, Zhang AJ, Pan WW, Xin QL, Chen JY, Zhang LL, et al. An IgD-Fc-Ig fusion protein restrains the activation of T and B cells by inhibiting IgD-IgDR-Lck signaling in rheumatoid arthritis. Acta Pharmacol Sin. 2022;43(2):387–400. 10.1038/s41401-021-00665-w PMC8791948 33864023

[12] Lin H, Zhao Z, Hao Y, He J, He J (2020). Long noncoding RNA HIF1A-AS2 facilitates cell survival and migration by sponging miR-33b-5p to modulate SIRT6 expression in osteosarcoma. Biochem Cell Biol.

[13] Uno Y, Yamazaki H (2020). Expression levels of microRNAs that are potential cytochrome P450 regulators in cynomolgus macaques. Xenobiotica.

[14] Chitu V, Pixley FJ, Macaluso F, Larson DR, Condeelis J, Yeung YG, Stanley ER (2005). The PCH family member MAYP/PSTPIP2 directly regulates F-actin bundling and enhances filopodia formation and motility in macrophages. Mol Biol Cell.

[15] Pixley FJ, Lee PS, Condeelis JS, Stanley ER (2001). Protein tyrosine phosphatase phi regulates paxillin tyrosine phosphorylation and mediates colony-stimulating factor 1-induced morphological changes in macrophages. Mol Cell Biol.

[16] Sztacho M, Segeletz S, Sanchez-Fernandez MA, Czupalla C, Niehage C, Hoflack B (2016). BAR Proteins PSTPIP1/2 Regulate Podosome Dynamics and the Resorption Activity of Osteoclasts. PLoS One.

[17] Xu JJ, Li HD, Du XS, Li JJ, Meng XM, Huang C, Li J (2021). Role of the F-BAR Family Member PSTPIP2 in Autoinflammatory Diseases. Front Immunol.

[18] Ferguson PJ, Bing X, Vasef MA, Ochoa LA, Mahgoub A, Waldschmidt TJ, Tygrett LT, Schlueter AJ, El-Shanti H (2006). A missense mutation in pstpip2 is associated with the murine autoinflammatory disorder chronic multifocal osteomyelitis. Bone.

[19] Alivernini S, MacDonald L, Elmesmari A, Finlay S, Tolusso B, Gigante MR, Petricca L, Di Mario C, Bui L, Perniola S (2020). Distinct synovial tissue macrophage subsets regulate inflammation and remission in rheumatoid arthritis. Nat Med.

[20] Culemann S, Gruneboom A, Nicolas-Avila JA, Weidner D, Lammle KF, Rothe T, Quintana JA, Kirchner P, Krljanac B, Eberhardt M (2019). Locally renewing resident synovial macrophages provide a protective barrier for the joint. Nature.

[21] Xu JJ, Zhu L, Li HD, Du XS, Li JJ, Yin NN, Meng XM, Huang C, Li J (2022). DNMT3a-mediated methylation of PSTPIP2 enhances inflammation in alcohol-induced liver injury via regulating STAT1 and NF-kappaB pathway. Pharmacol Res.

[22] Chitu V, Nacu V, Charles JF, Henne WM, McMahon HT, Nandi S, Ketchum H, Harris R, Nakamura MC, Stanley ER (2012). PSTPIP2 deficiency in mice causes osteopenia and increased differentiation of multipotent myeloid precursors into osteoclasts. Blood.

[23] Ferguson PJ, Laxer RM (2015). New discoveries in CRMO: IL-1beta, the neutrophil, and the microbiome implicated in disease pathogenesis in Pstpip2-deficient mice. Semin Immunopathol.

[24] Garcia-Rojas MD, Palma-Cordero G, Martinez-Ramirez CO, Ponce de Leon-Suarez V, Valdes-Flores M, Castro-Hernandez C, Rubio-Lightbourn J, Hernandez-Zamora E, Reyes-Maldonado E, Velazquez-Cruz R, et al. Association of Polymorphisms in Estrogen Receptor Genes (ESR1 and ESR2) with Osteoporosis and Fracture-Involvement of Comorbidities and Epistasis. DNA Cell Biol. 2022;41(4):437–46. 10.1089/dna.2021.1165 35285722

[25] Steppe L, Bulow J, Tuckermann J, Ignatius A, Haffner-Luntzer M. Bone Mass and Osteoblast Activity Are Sex-Dependent in Mice Lacking the Estrogen Receptor alpha in Chondrocytes and Osteoblast Progenitor Cells. Int J Mol Sci. 2022;23(5):2902. 10.3390/ijms23052902 PMC8911122 35270044

[26] Sun Y, Leng P, Guo P, Gao H, Liu Y, Li C, Li Z, Zhang H (2021). G protein coupled estrogen receptor attenuates mechanical stress-mediated apoptosis of chondrocyte in osteoarthritis via suppression of Piezo1. Mol Med.

[27] Taheri M, Shoorei H, Dinger ME, Ghafouri-Fard S. Perspectives on the Role of Non-Coding RNAs in the Regulation of Expression and Function of the Estrogen Receptor. Cancers (Basel). 2020;12(8):2162. 10.3390/cancers12082162 PMC7465269 32759784

