# Supplementary material for: PSTPIP2 regulates synovial macrophages polarization and dynamics via ERβ in the joint microenvironment
Source: Arthritis Res Ther. 2022 Nov 2;24:247. doi: 10.1186/s13075-022-02939-y (PMC9628044; doi:10.1186/s13075-022-02939-y)
Supplement: Supplementary file 1 — Additional file 1: Table 1. Clinical information of the participants (mean ± SD). Supplementary Figure 1. (A) RF levels in RA patients. (B) ASO levels in RA patients. (C) Scatter plot (left) and histogram (right) of CD11b+CD86+ cells in peripheral blood. (D) Scatter plot (left) and histogram (right) of CD11b+CD86+ cells in synovial tissue. (E) Correlation analysis of the level of PSTPIP2 in CD11b+ monocytes with CD11b+CD86+ cells in peripheral blood. (F) Correlation analysis of the level of PSTPIP2 in CD11b+ monocytes with CD11b+CD86+ cells in synovial tissue. P < 0.05 indicates that the difference is statistically significant. (G) Peripheral blood monocyte/macrophage gating strategy. (H) Synovial tissue monocyte/macrophage gating strategy. Data represent mean ± SD (unpaired t test for C and D; linear regression for E and F). Supplementary Figure 2. (A) Scatter plot of ZO-1 expression in F4/80+PSTPIP2+ synovial macrophages on day35. (B) Scatter plot of ZO-1 expression in F4/80+PSTPIP2+ synovial macrophages on day70. (C) Histogram of F4/80+PSTPIP2+ZO-1+ synovial macrophages in synovial tissue on day35 and day70. P < 0.05 indicates that the difference is statistically significant. Data represent mean ± SD (unpaired t test for C). Supplementary Figure 3. (A) Fold plot of arthritis score over time in CIA mice. (B) Photographs of the paws of mice at different time points. (C) HE staining of the knee in mice at different time points. (D) Expression of PSTPIP2 in F4/80+ macrophages at different time points in synovial tissue. (E) Immunofluorescence staining of PSTPI2P in synovial tissues at different time points. Green: PSTPIP2, Blue: DAPI. (F) Correlation analysis of PSTPIP2 levels in F4/80+ synovial macrophages with arthritis scores in the CIA model. P < 0.05 indicates that the difference is statistically significant. Data represent mean ± SD (one-way ANOVA for D; linear regression for F). Supplementary Figure 4. (A) SafraninO-fast green staining of the knee joint. (B) T [file 13075_2022_2939_MOESM1_ESM.docx]

**Supplementary figure**

**Table 1. Clinical information of the participants (mean ± SD).**

| **Participants, n=35** | **Acute trauma =5** | **Rheumatoid arthritis=23** | **p value** |
| --- | --- | --- | --- |
| Age (yeas) | 55.8 ± 4.97 | 53.13 ± 7.051 | 0.443 |
| Weight (kg) | 62.00 ± 7.00 | 65.06 ± 5.859 | 0.340 |
| BMI (kg/m^2^) | 24.60 ± 0.579 | 24.34 ± 0.726 | 0.471 |

**Supplementary figures and legends**


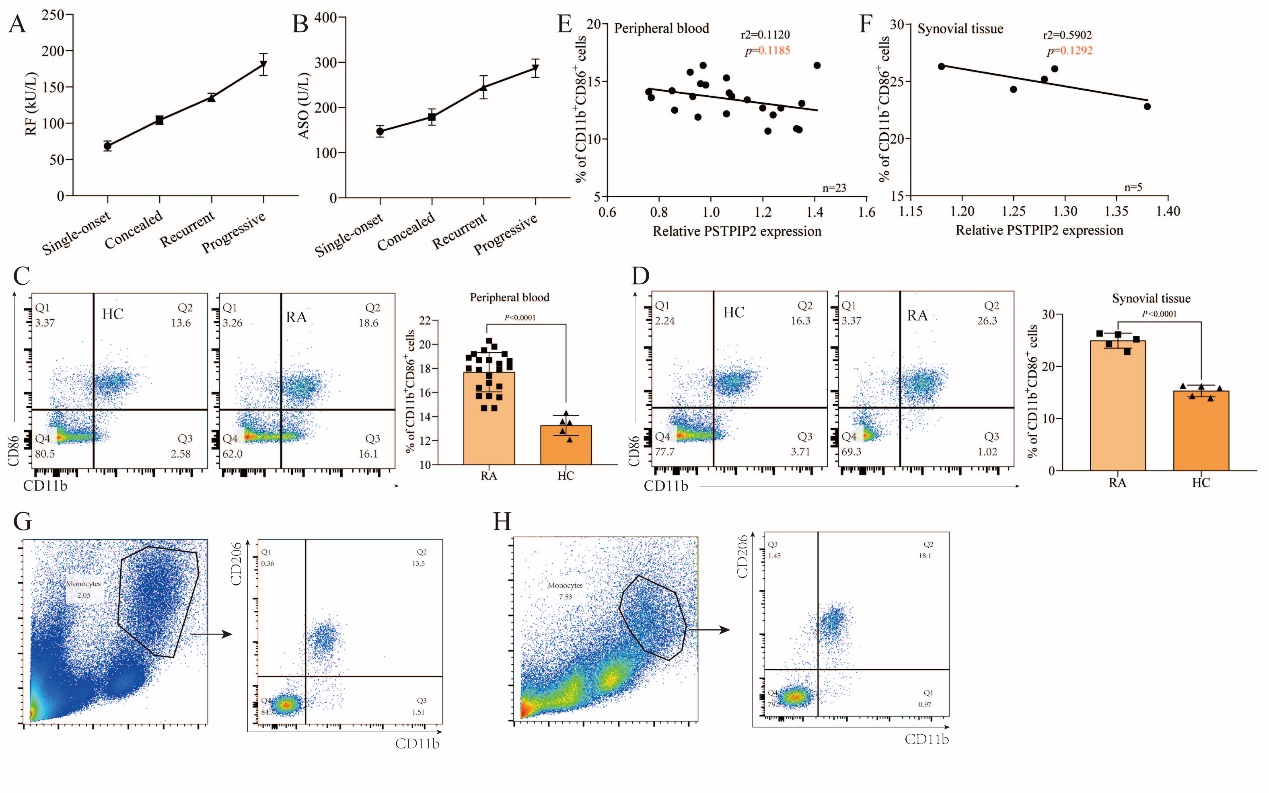


**Supplementary figure 1**. (A) RF levels in RA patients. (B) ASO levels in RA patients. (C) Scatter plot (left) and histogram (right) of CD11b^+^CD86^+^ cells in peripheral blood. (D) Scatter plot (left) and histogram (right) of CD11b^+^CD86^+^ cells in synovial tissue. (E) Correlation analysis of the level of PSTPIP2 in CD11b^+^ monocytes with CD11b^+^CD86^+^ cells in peripheral blood. (F) Correlation analysis of the level of PSTPIP2 in CD11b^+^ monocytes with CD11b^+^CD86^+^ cells in synovial tissue. *P* < 0.05 indicates that the difference is statistically significant. (G) Peripheral blood monocyte/macrophage gating strategy. (H) Synovial tissue monocyte/macrophage gating strategy. Data represent mean ± SD (unpaired t test for C and D; linear regression for E and F).


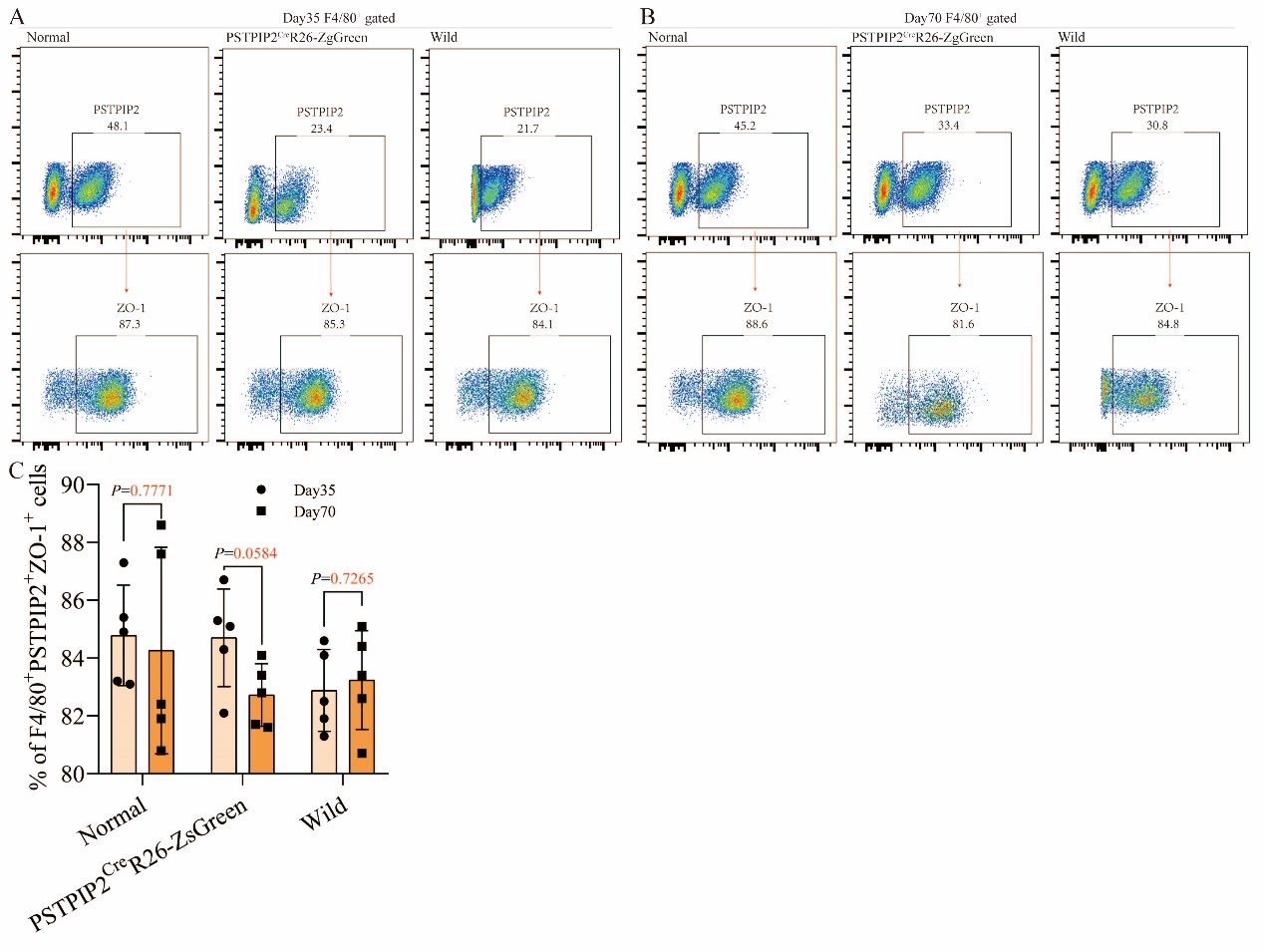


**Supplementary figure 2**. (A) Scatter plot of ZO-1 expression in F4/80^+^PSTPIP2^+^ synovial macrophages on day35. (B) Scatter plot of ZO-1 expression in F4/80^+^PSTPIP2^+^ synovial macrophages on day70. (C) Histogram of F4/80^+^PSTPIP2^+^ZO-1^+^ synovial macrophages in synovial tissue on day35 and day70. *P* < 0.05 indicates that the difference is statistically significant. Data represent mean ± SD (unpaired t test for C).


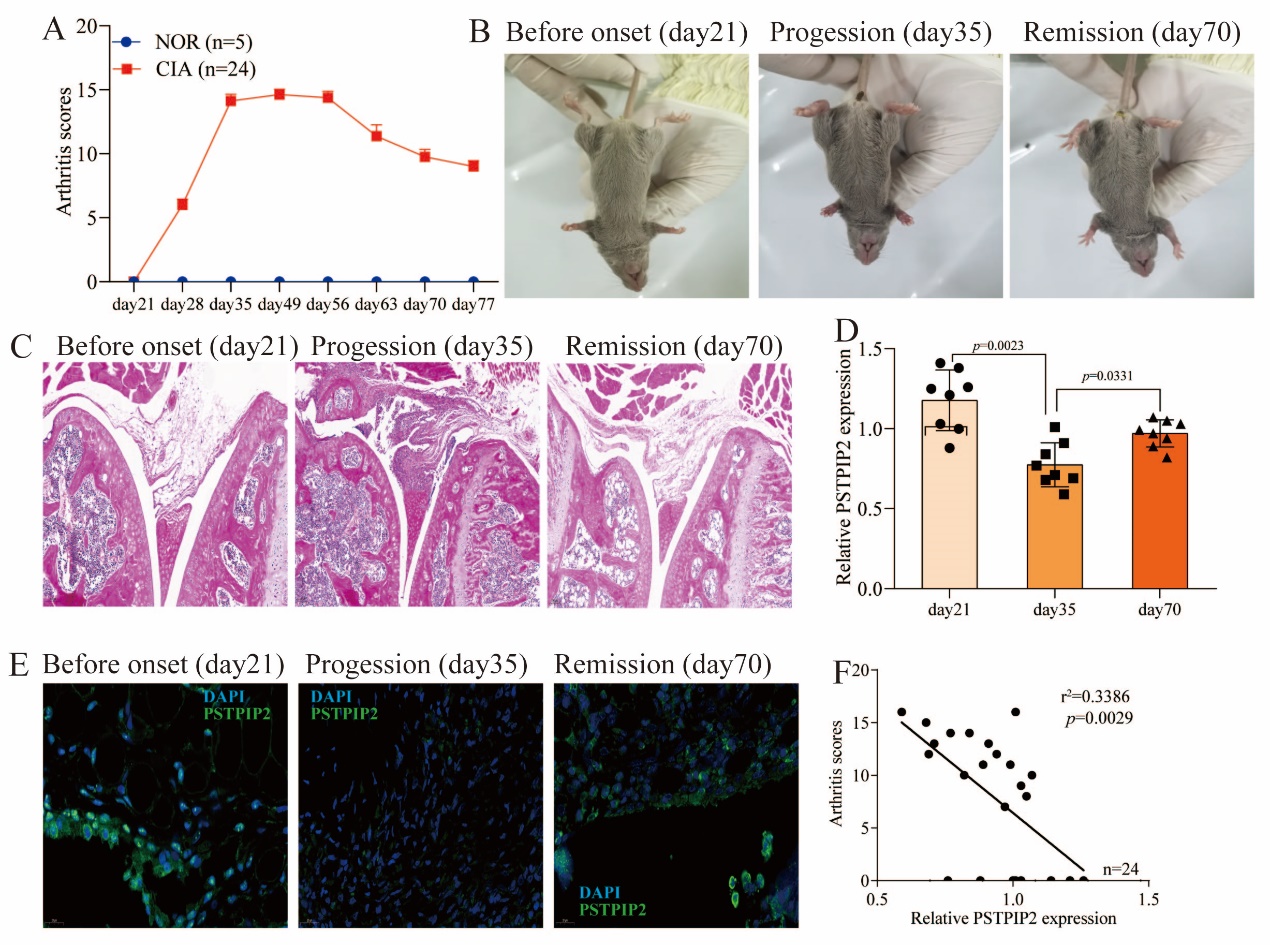


**Supplementary figure 3**. (A) Fold plot of arthritis score over time in CIA mice. (B) Photographs of the paws of mice at different time points. (C) HE staining of the knee in mice at different time points. (D) Expression of PSTPIP2 in F4/80^+^ macrophages at different time points in synovial tissue. (E) Immunofluorescence staining of PSTPI2P in synovial tissues at different time points. Green: PSTPIP2, Blue: DAPI. (F) Correlation analysis of PSTPIP2 levels in F4/80^+^ synovial macrophages with arthritis scores in the CIA model. *P* < 0.05 indicates that the difference is statistically significant. Data represent mean ± SD (one-way ANOVA for D; linear regression for F).


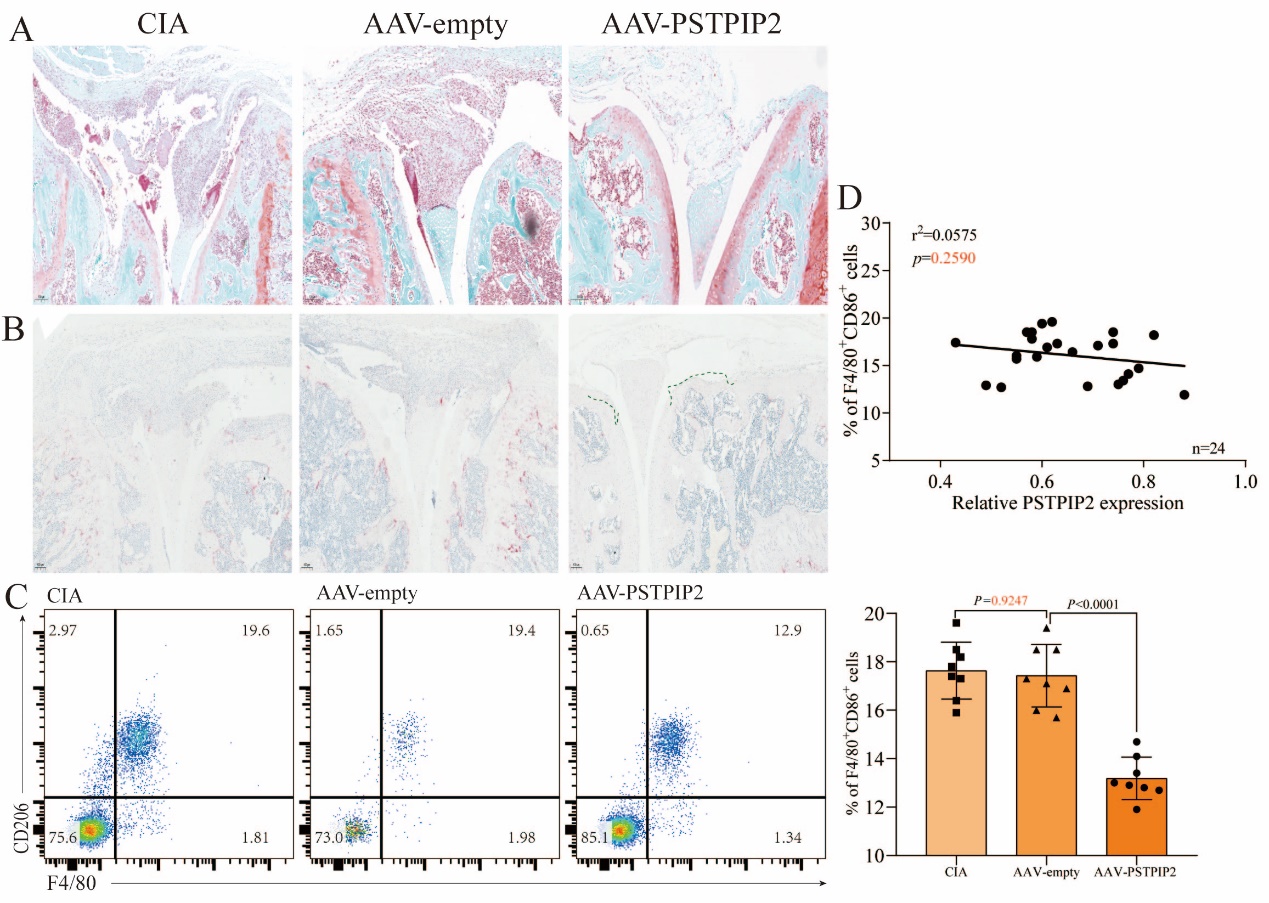


**Supplementary figure 4**. (A) SafraninO-fast green staining of the knee joint. (B) TRAP staining of the knee joint. (C) Analysis of synovial macrophages polarization: the percentage of F4/80^+^CD86^+^ cells in synovial tissue. Scatter plot on the left, histogram on the right. (D) Correlation analysis of PSTPIP2 level in F4/80^+^ synovial macrophages with the frequency of F4/80^+^CD86^+^ cells in synovial tissue. *P* < 0.05 indicates that the difference is statistically significant. Data represent mean ± SD (one-way ANOVA for C; linear regression for D).


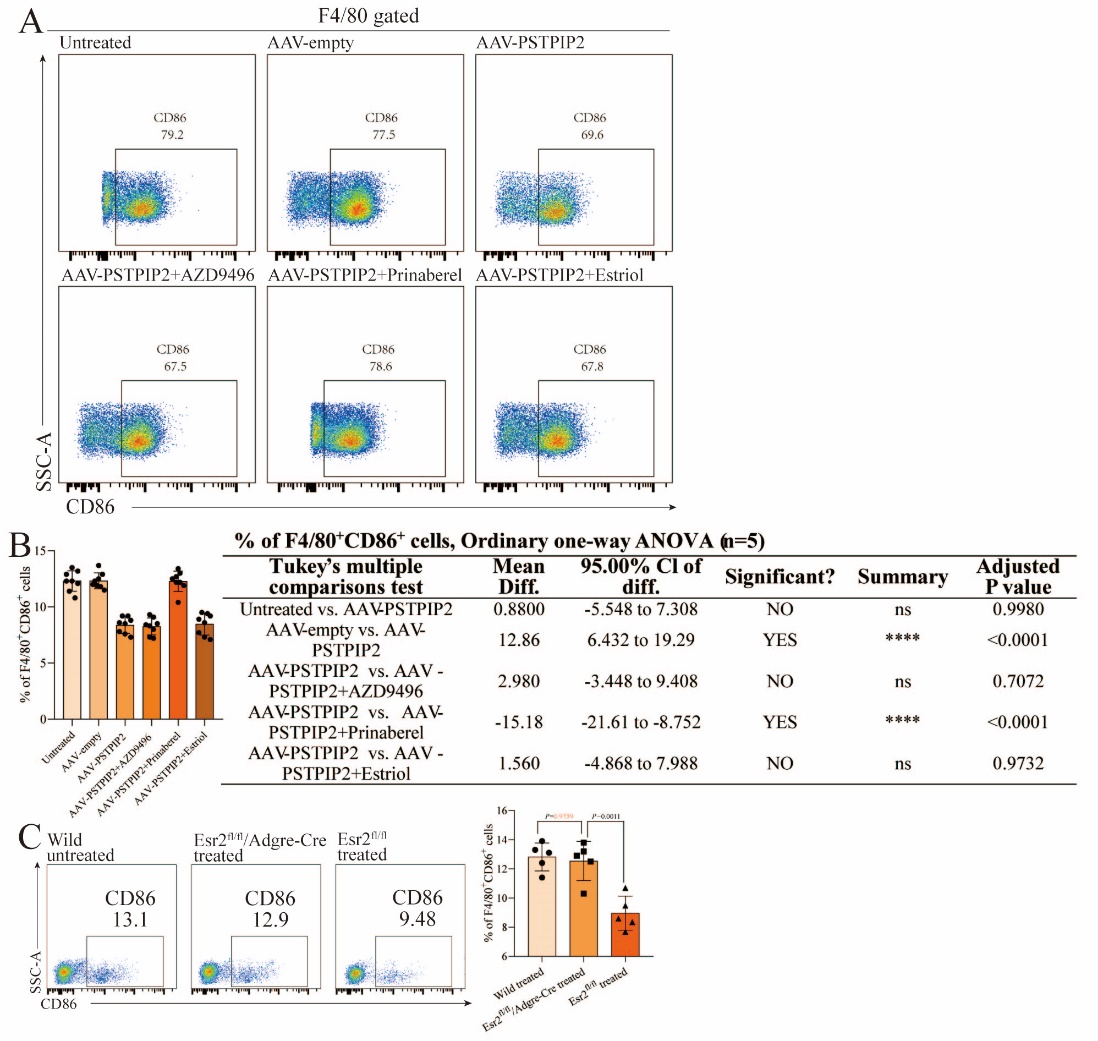


**Supplementary figure 5**. (A) Effect of estrogen receptors on macrophage polarization *in vitro*: Scatter plot of F4/80^+^CD86^+^ cells. AZD9496: selective ERα antagonist, Prinaberel: selective ERβ antagonist, Estriol: G protein-coupled estrogen receptor antagonist. (B) Histogram of F4/80^+^CD86^+^ cells (left). One-way ANOVA data for F4/80^+^CD206^+^ cells (right). (C) Analysis of synovial macrophages polarization: the percentage of F4/80^+^CD86^+^ cells in synovial tissue. Scatter plot on the left, histogram on the right. *P* < 0.05 indicates that the difference is statistically significant. Data represent mean ± SD (one-way ANOVA for B and C).
